# Adaptation and validation of the Treatment Burden Questionnaire (TBQ) in English using an internet platform

**DOI:** 10.1186/1741-7015-12-109

**Published:** 2014-07-02

**Authors:** Viet-Thi Tran, Magdalena Harrington, Victor M Montori, Caroline Barnes, Paul Wicks, Philippe Ravaud

**Affiliations:** 1 Department of General Medicine, Paris Diderot University, Paris, France; 2 METHODS Team, Epidemiology and Statistics Sorbonne Paris Cité Research Centre, UMR 1153, INSERM, 1 place du Parvis Notre-Dame, Paris 75181, France; 3 Centre d'Épidémiologie Clinique, HôpitalHôtel-Dieu, Assistance Publique-Hôpitaux de Paris, Paris, France; 4 PatientsLikeMe, Inc., Cambridge, Massachusetts, USA; 5 Division of Health Care and Policy Research, Department of Health Sciences Research and Knowledge and Evaluation Research Unit, Mayo Clinic, Rochester, MN, USA; 6 Paris Descartes University, Paris, France; 7 Department of Epidemiology, Columbia University Mailman School of Public Health, New York, NY, USA

**Keywords:** Chronic Disease/therapy, Patient Participation, Quality of Life, Medication Adherence, Questionnaires

## Abstract

**Background:**

Treatment burden refers to the workload imposed by healthcare on patients, and the effect this has on quality of life. The Treatment Burden Questionnaire (TBQ) aims to assess treatment burden in different condition and treatment contexts. Here, we aimed to evaluate the validity and reliability of an English version of the TBQ, a scale that was originally developed in French.

**Methods:**

The TBQ was translated into English by a forward-backward translation method. Wording and possible missing items were assessed during a pretest involving 200 patients with chronic conditions. Measurement properties of the instrument were assessed online with a patient network, using the PatientsLikeMe website. Dimensional structure of the questionnaire was assessed by factor analysis. Construct validity was assessed by associating TBQ global score wıth clinical variables, adherence to medication assessed by Morisky’s Medication Adherence Scale (MMAS-8), quality of life (QOL) assessed by the PatientsLikeMe Quality of Life Scale (PLMQOL), and patients’ confidence in their knowledge of their conditions and treatments. Reliability was determined by a test–retest method.

**Results:**

In total, 610 patients with chronic conditions, mainly from the USA, UK, Canada, Australia, or New Zealand, completed the TBQ between September and October 2013. The English TBQ showed a unidimensional structure with Cronbach α of 0.90. The TBQ global score was negatively correlated with the PLMQOL score (r_s_ = −0.50; p < 0.0001). Low rather than moderate or high adherence to medication was associated with high TBQ score (mean [SD] TBQ score 61.8 [30.5] vs. 37.7 [27.5]; *P* < 0.0001). The treatment burden was higher for patients who had insufficient knowledge compared with those who had sufficient knowledge about their treatments (mean ± SD TBQ score 62.3 ± 31.3 vs. 47.8 ± 30.4; *P* < 0.0001) and conditions (63.0 ± 31.6 vs. 49.3 ± 30.7; *P* < 0.0001). The intraclass correlation coefficient for the retest (n = 282) was 0.77 (95% CI 0.70 to 0.82).

**Conclusions:**

We found that the English TBQ is a reliable instrument in this population, and provide evidence supporting the construct validity for its use to assess treatment burden for patients with one or more chronic conditions in English-speaking countries.

## Background

Treatment burden is defined as the ‘work’ of being a patient and its effect on the quality of life (QOL) of patients. It represents the challenges associated with everything patients have to do to take care of themselves (e.g. medication intake, drug management, self-monitoring, visits to the physician, laboratory tests, lifestyle changes, administrative tasks to access and coordinate care) [1-3]. This work can represent a tremendous investment of time, attention, cognitive energy, and effort. For example, a patient with a chronic condition may spend 2 hours or more per day on health-related activities [4], and this increases for patients with multiple chronic conditions who receive treatment or recommendations for each of their diseases [5,6]. For these patients, treatment burden could be considered a crucial outcome for disease management [7]. Difficulties with their treatment are often not shared in depth by patients during medical consultations [8], and physicians are often not aware of the challenges their patients face in coping with everything asked of them [9]. Thus, a tool that is simple to understand and easy to administer is needed to identify overburdened patients in daily practice and for research, in order to design new therapeutic strategies that are both efficient and acceptable for patients.

The Treatment Burden Questionnaire (TBQ) is the only existing instrument that measures treatment burden without restricting its scope to a single condition or treatment context [9,10]. The TBQ is composed of 13 items rated on a Likert scale ranging from 0 (not a problem), to 10 (big problem). The TBQ was derived from a literature review and qualitative semi-structured interviews with patients in France. It assesses the burden associated with taking medicine, self-monitoring, laboratory tests, doctor visits, need for organization, administrative tasks, following advice on diet and physical activity, and social impact of the treatment. Item scores can be summed into a global score, ranging from 0 to 130. The instrument was first developed with a sample of patients with one or more chronic conditions, who were recruited from hospitals and general-practice clinics in France.

To measure treatment burden in English-speaking countries, the TBQ needs to be adapted: items must remain comprehensible without changing their original meaning, and it should be recognized that new items could arise in different contexts whereas some existing items might be irrelevant. Patients must be involved in the process to ensure that the resulting tool is clear and relevant [11].

In this study, we aimed to assess the validity and reliability of an English version of the TBQ intended to be used in both routine practice and in research for patients with at least one chronic condition living in the USA, Canada, UK, Australia, or New Zealand.

## Methods

Adaptation and validation of the TBQ in English followed a multi-step approach recommended in the literature [12-14]: 1) review the conceptual evidence about treatment burden in English-speaking countries; 2) translate the TBQ into English; 3) pretest the instrument with patients to assess the relevance of items, clarity and wording and; 4) assess validity and reliability by a test–retest of the adapted instrument.

### Conceptual evidence of treatment burden in English-speaking countries

During a literature review of MEDLINE via Pubmed, we identified several articles describing the concept of treatment burden in English-speaking countries. In the USA, Eton and colleagues identified three broad themes for treatment burden: 1) work patients must do to take care of their health, 2) problem-focused strategies to facilitate self-care, and 3) factors that exacerbate the perceived burden [2]. In the UK, Gallacher and associates found four similar themes in patients with chronic heart failure and stroke: 1) learning about treatments and their consequences, 2) engaging with others, 3) adhering to treatment and lifestyle changes, and 4) monitoring their treatments [15,16]. Finally, in Australia, Sav and associates identified four inter-related components of treatment burden: 1) financial, 2) time and travel, 3) medication, and 4) healthcare access burdens [17].

The original TBQ encompassed all of these domains, with the exception of the financial treatment burden, because in France, the public health insurance program guarantees healthcare free of charge for patients with chronic conditions. Therefore, we added a new item in the English adaptation of the TBQ: ‘How would you rate the financial burden associated with your healthcare (e.g., out-of-pocket expenses or expenses not covered by insurance)?’

### Translation of the TBQ into English

Translation of the TBQ intoEnglish involved a classic ‘forward-backward’ translation method [13]. First, the original instrument in French was translated into English by two bilingual translators; one (CB) had a medical background, and was familiar with the concept of treatment burden. Second, the two obtained translations were synthesized and reviewed by a committee, which included the authors of the original questionnaire. Third, two different translators, blinded to the original version, back-translated the questionnaire into French. Finally, the committee reviewed and synthesized all translations to elaborate English items that were similar to the original items and easy to answer.

### Pre-testing the instrument

To assess the relevance of items, clarity, and wording, we pre-tested the obtained instrument with a convenience sample of 200 participants in August 2013 (see Additional file 1). We used an internet platform, the Open Research Exchange (ORE) [18,19], to recruit patients on PatientsLikeMe (PLM) [20], an online network where 200,000 voluntary participants with chronic conditions share data about their treatment, conditions, and symptoms. Members of PLM join the site with the expectation that they will be participating in research. To participate, patients had to have at least one chronic condition (defined as requiring ongoing healthcare for at least 6 months). After having answered the questionnaire, they provided feedback about 1) the clarity, wording, and relevance of the items, and 2) any burden that they felt was not covered or insufficiently covered in the questionnaire in an open-ended manner. Their answers were categorized and discussed by two of the authors (VT-T and CB).

Concerning the wording of items, 10 patients (5.0%) felt that the word ‘constraints’ was confusing, thus we replaced the word ‘constraints’ by the word ‘problem’, as suggested by the patients. Patients were also asked whether there were any important elements of treatment burden that they considered to be missing from questions: 15 patients (7.5%) thought that relationships between patients and healthcare providers were insufficiently covered in the original items. Other suggestions were either specific to a particular condition, were related to the burden of disease, or were already covered in the existing items. Thus, we added a new item for testing: ‘How would you rate the difficulties you could have in your relationships with healthcare providers (e.g., feeling not listened to enough or not taken seriously)?’ After the pretest, the English TBQ was therefore composed of 15 items, with rating scales ranging from 0 to 10 and labeled anchors (‘not a problem’ and ‘large problem’).

### Assessment of validity and reliability of the English TBQ

We studied the measurement properties of the instrument by 1) describing the item properties, 2) assessing factor structure, 3) assessing construct validity, and 4) assessing reliability by test–retest.

We recruited a convenience sample of patients via the aforementioned internet platform. Patients were eligible if they were 18 years or older, and had at least one condition that had required ongoing health care for at least 6 months. We sent email invitations to a random sample of 3,000 members on the internet platform who did not participate in the pretest and who met the eligibility criteria, encouraging them to connect to the website and complete the questionnaire. To increase the number of respondents, an email reminder was sent after 2 months. Patients consented electronically to participate in the study. The recruitment message outlined the purpose of the study and reminded patients that they were under no obligation to participate, that their aggregated results may be published. Because there were no anticipated adverse consequences for participation, institutional review board (IRB) approval was not sought for this project.

Item properties were described using three criteria: 1) proportion of missing answers, 2) relevance of items assessed by the proportion of ‘does not apply,’ and 3) score distributions.

Factor structure was investigated by exploratory factorial analysis. Scree plots were used to visualize a break between factors with large eigenvalues and those with smaller eigenvalues. Factors that appeared before the horizontal break were assumed to be meaningful. Internal consistency was assessed by Cronbach’s α [21], and was considered acceptable between 0.70 and 0.95 [22].

The global score of the TBQ (TBQ Global score) was the sum of the answers to each item. ‘Does not apply’ or missing answers were considered the lowest possible score (0) because we considered that a patient not concerned by a domain of treatment burden had no burden for that domain.

Construct validity was tested by confirming four pre-specified hypotheses. First, we expected a negative correlation between treatment burden (as measured by the TBQ global score) by the TBQ global score) and quality of life. Quality of life was measured by the PatientsLikeMe Quality of Life (PLMQOL) scale, a validated 24-item questionnaire assessing physical, mental, and social quality of life. PLMQOL scores range from 0 to 100 for each domain (higher scores indicating better quality of life) and are summed for a global assessment of quality of life [23]. Second, we predicted an association between TBQ global score and adherence to medication: the greater the treatment burden, the lower the adherence to treatment. Adherence to medical treatment was measured by Morisky’s Medication Adherence Scale 8 (MMAS-8) [24,25], a validated eight-item questionnaire, with scores ranging from 0 to 8. High adherence is a score of 8; medium adherence, 6 to 7; and low adherence, less than 6 [24]. Third, we hypothesized that patients with better knowledge of their conditions and treatments would have a low treatment burden. Confidence in patients’ knowledge about their treatments and conditions was assessed by two questions: ‘Do you think you have sufficient knowledge about your conditions (e.g., symptoms, disease progression)?’ and ‘Do you think you have sufficient knowledge about your treatments (e.g., possible side effects, expected benefits, other treatment options)?’. Answers were rated on a five-step scale: ‘very sufficient’, ‘sufficient’, ‘average’, ‘insufficient’ and ‘very insufficient’. Finally, we assumed a positive correlation between TBQ global score and the following clinical variables: 1) number of conditions, 2) drug administration (number of tablets, injections, and administrations per day), and 3) medical follow-up (number of different physicians, medical appointments per month, and hospitalizations per year).

To elicit the chronic conditions a patient had, we asked the patient to self identify the condition(s) from a list recommended as core for any measure of multimorbidity [26]. Options were presented as categories illustrated by common conditions; for example: ‘Rheumatologic disease (e.g. osteoporosis, arthritis, or inflammatory polyarthropathies)’. Patients were encouraged to complete their answer with free text. The text was analyzed, and the condition was categorized in the appropriate category by a single investigator (VTT).

The association between the TBQ global score, quality of life score and clinical variables was assessed by Spearman correlation coefficient (r_s_), which was considered high when greater 0.50, and moderate when 0.35 to 0.50 [27]. Wilcoxon and Kruskal-Wallis tests were used to compare qualitative variables across groups. *P* < 0.05 was considered statistically significant.

Reliability of the instrument was determined by a test–retest method. Patients were asked to complete the new instrument twice: at baseline and again after 2 weeks when they received an email reminder. Reliability was assessed by the intraclass correlation coefficient (ICC) for agreement [28], defined as the ratio of the subject variance by the sum of the subject variance, the rater variance and the residual. The 95% confidence interval (CI) was determined by a bootstrap method. Agreement was considered acceptable when ICC was greater than 0.60 [29]. Agreement was represented by Bland and Altman plots, which represent the differences between two measurements against the means of the two measurements [30].

Statistical analyses were performed using SAS (version 9.3; SAS Inst., Cary, NC, USA) and R (version 3.0 [31], the R Foundation for Statistical Computing, Vienna, Austria).

## Results

In total, 3,000 patients were invited to complete the TBQ, and 610 (20.3%) did so between September and October 2013. The mean ± SD age was 51.5 ± 2.4 years. Patients resided mainly in the USA (57.5%), Canada (8.4%), UK (8.7%), and Australia/New Zealand (3.4%). Some patients (13.3%) lived in other countries (Belgium, Brazil, Chile, Czech Republic, Denmark, Finland, France, Germany, India, Ireland, Israel, Italy, Malaysia, Norway, Oman, Portugal, Russia, South Africa, Spain, Switzerland, Ukraine, and Uzbekistan), or the country of residence was missing (8.7%). The mean ± SD number of chronic conditions was 2.9 ± 1.9 (range 1 to 11) (Table 1).

**Table 1:** Demographic and clinical characteristics of patients in the validation study (n = 610) and retest of the Treatment Burden Questionnaire (TBQ) for an English-speaking population (n = 282)

**Characteristic**	**Validation study(n = 610)**	**Retest(n = 282)**
Age, years^a^	51.5 ± 12.4	52.3 ± 12.3
Female sex, n (%)	451 (76.7)	215 (76.2)
Country of residence, n (%)		
USA	351 (57.5)	172 (61.0)
Canada	51 (8.4)	20 (7.1)
UK	53 (8.7)	26 (9.2)
Australia/New Zealand	21 (3.4)	10 (3.5)
Other/missing	134 (22.0)	54 (19.1)
Educational level, n(%)		
Less than High School	24 (3.9)	10 (3.5)
High school graduate or General Education Diploma	99 (16.2)	55 (19.5)
Some College	220 (36.1)	95 (33.7)
Bachelor’s degree	134 (22.0)	60 (21.3)
Graduate degree	124 (20.3)	58 (20.6)
Treatments, n		
Tablets and pills/day	8.5 ± 6.4	8.6 ± 6.7
Injections/week	1.4 ± 4.6	1.5 ± 4.1
Drug administrations/day	3.0 ± 2.0	2.9 ± 1.9
Different doctors the patient sees	3.0 ± 2.3	2.9 ± 1.5
Appointments/month	2.9 ± 2.9	2.7 ± 2.7
Hospitalizations/year	0.5 ± 1.7	0.5 ± 1.1
Presence of an informal caregiver, n (%)	280 (45.9)	125 (44.3)
Most common location for medical consultations, n (%)		
Public hospital	63 (10.3)	30 (10.6)
Private hospital	20 (3.3)	4 (1.4)
General practice clinic	291 (47.7)	135 (47.9)
Specialist clinic	224 (36.7)	106 (37.6)
Duration of oldest chronic condition, years, n (%)		
<5	182 (29.8)	75 (26.6)
5 to 10	217 (35.6)	108 (38.3)
> 10	205 (33.6)	96 (34.0)
Chronic conditions, n	2.9 ± 1.9	2.9 ± 1.9
Conditions, n (%)^b^		
Neurologic disease	277 (45.4)	134 (47.5)
Psychiatric disease	250 (41.0)	107 (37.9)
Rheumatologic disease	203 (33.3)	89 (31.6)
High blood pressure	156 (25.6)	62 (22.0)
Gastrointestinal disease	129 (21.1)	67 (23.7)
Endocrine disorder (other than diabetes)	121 (19.8)	53 (18.8)
Lung disease	93 (15.2)	45 (16.0)
Vision problems	83 (13.6)	35 (12.4)
Fibromyalgia	79 (12.9)	30 (10.6)
Skin disease	71 (11.6)	33 (11.7)
Hearing problem	49 (8.0)	27 (9.6)
Diabetes	45 (7.4)	19 (6.7)
Kidney disease	38 (6.2)	12 (4.2)
Heart disease	34 (5.6)	14 (5.0)
Cancer or malignant blood disease	31 (5.1)	19 (6.7)
Infectious disease	19 (3.1)	10 (3.5)
Stroke or cerebrovascular disease	17 (2.8)	8 (2.8)

^a^Data are mean ± SD unless otherwise indicated.

^b^Any given patient can have multiple conditions.

We found a high completion rate (>99%) for all items. All items were relevant for all patients, with the proportion of ‘does not apply’ ranging from 0.6% to 7.2%, with the exception of the burden associated with self-monitoring (30.3%) (see Additional file 2). The TBQ global score was the sum of the answers to each of the 15 items, and ranged from 0 to 150 (‘Does not apply’ was considered the lowest possible score). The global score had a high correlation with the score for each item of the TBQ (r_s_ = 0.47 to 0.71) (see Additional file 3).

Factor structure, assessed by scree plots, favored a unidimensional instrument. The first factor explained 87% of the variance, and had an eigenvalue of 5.91. For subsequent factors, eigenvalues wereless than 0.80 (see Additional file 4).

Construct validity showed a significant moderate negative correlation between the TBQ global score and PLMQOL score (r_s_ = −0.50; *P* < 0.0001). Correlation coefficients ranged from r_s_ = −0.39 (*P* < 0.0001) for physical quality of life to r_s_ = −0.50 (*P* < 0.0001) for mental quality of life, indicating that patients with high TBQ score had low quality of life (Table 2). We found a significant association between treatment burden measured by the TBQ global score and adherence to medication measured by the MMAS-8: mean ± SD TBQ global score was 37.7 ± 27.5 for patients with high or moderate adherence, and 61.8 ± 30.5 for patients with low adherence (*P* < 0.0001) (Table 2). The TBQ score was also significantly associated with patients’ confidence in their knowledge about their treatments and conditions. The mean ± SD TBQ global score was 47.8 ± 30.4 for patients who felt that they had sufficient knowledge about their treatment, versus 62.3 ± 31.3 for those who felt that they had insufficient knowledge (*P* < 0.0001). The mean ± SD TBQ global score was 49.3 ± 30.7 for patients declaring sufficient knowledge about their conditions versus 63.0 ± 31.6 for those with insufficient knowledge (*P* < 0.0001) (Table 2). Finally, we found a low to moderate significant positive correlation of TBQ score with clinical variables in terms of number of different conditions, drug administrations (number of tablets, injections, and administrations per day) and medical follow-up (number of different physicians, medical appointments per month, and hospitalizations per year) (see Additional file 5).

**Table 2:** Association of adherence to medication, patients’ evaluation of their knowledge about their treatment and conditions and QOL with TBQ global score (n = 610)

	**TBQ global score, mean ± SD**	**Spearman correlation coefficient**	***P*** **value**
Adherence to treatment assessed by the MMAS-8^a^			
High and moderate adherence	37.7 ± 27.5	–	<0.0001
Low adherence	61.8 ± 30.5	–	<0.0001
Patients’ evaluation of their knowledge of:			
Their treatments^b^			
Sufficient	47.8 ± 30.4	–	<0.0001
Insufficient	62.3 ± 31.3	–	<0.0001
Their conditions^b^			
Sufficient	49.3 ± 30.7	–	<0.0001
Insufficient	63.0 ± 31.6	–	<0.0001
Quality of life assessed by the PLMQOL^c^			
Physical QOL	−	−0.39	<0.0001
Mental QOL	−	−0.50	<0.0001
Social QOL	−	−0.49	<0.0001
Overall assessment of QOL	−	−0.50	<0.0001

PLMQOL, PatientsLikeMe Quality of Life; QOL, quality of life; TBQ, Treatment Burden Questionnaire.

^a^High adherence is a score of 8; medium adherence, 6 to 7; and low adherence, <6 [24]. ^b^‘Very sufficient’ and ‘sufficient’ was considered sufficient and ‘average’, ‘insufficient’ and ‘very insufficient’was considered insufficient.

^c^PLMQOL assesses physical, mental and social QOL; high scores indicate better QOL, while negative correlation coefficients indicate that patients with high treatment burden have low QOL.

**Figure 1:**
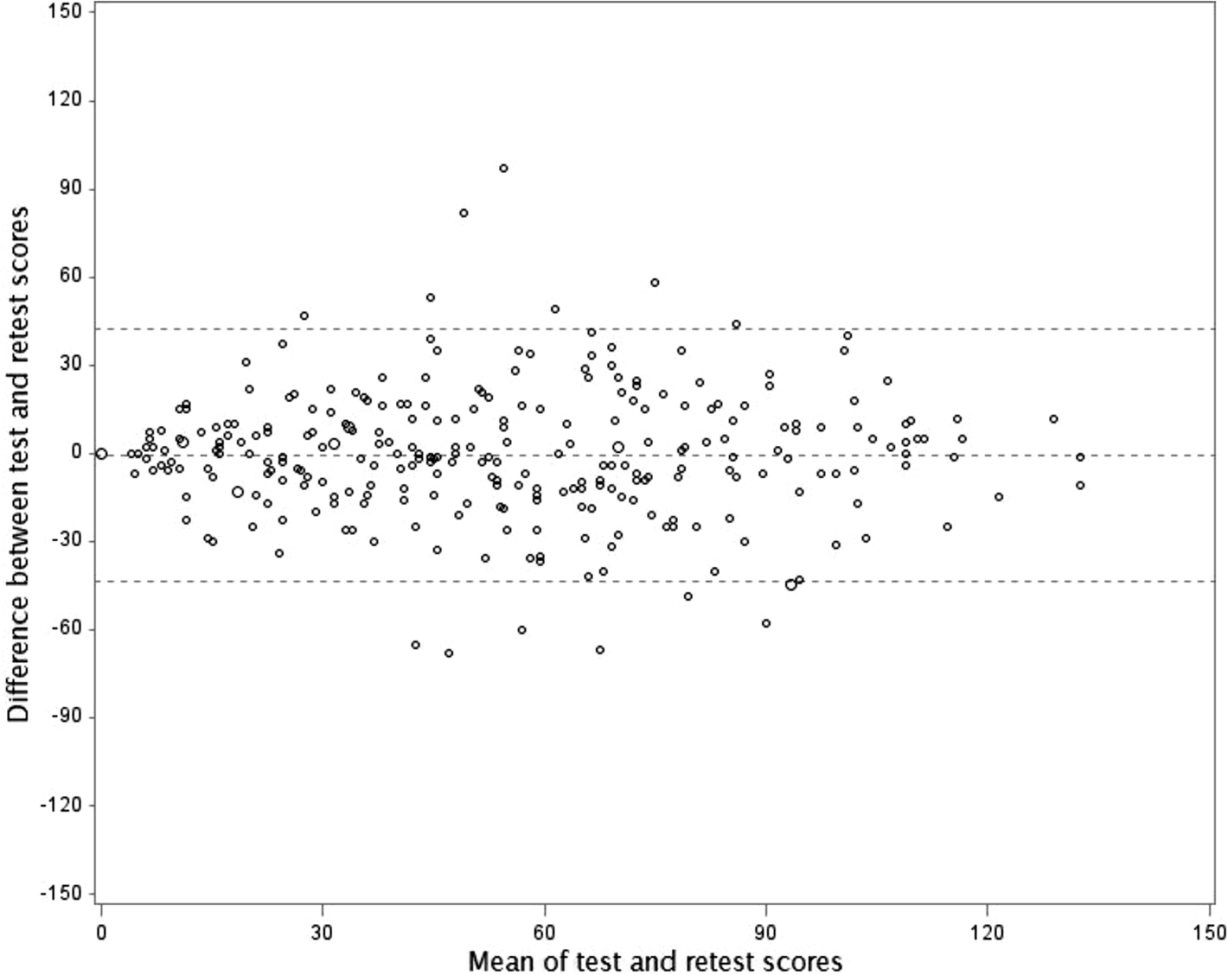
Bland and Altman plot of the test–retest reliability of the TBQ global score (n = 280).

Reliability was assessed by a test–retest method for 282 patients (46%). Participants in the retest showed similar characteristics as patients in the test phase (Table 1). For the TBQ global score, the ICC for agreement was 0.77 (95% CI 0.70 to 0.82) (see Additional file 6). Bland and Altman plots for the global TBQ score showed a mean difference of −0.58; 95% limits of agreement −43.5 and 42.3 (Figure 1).

**Figure 2:**
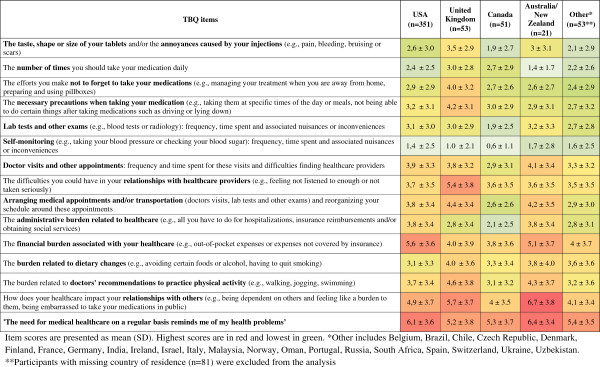
**Comparison of scores for TBQ items by country (n = 529)**^**a**^**.**

Subgroup analysis showed a high TBQ global score for patients who needed equipment such as wheelchairs or canes (mean ± SD score 69.0 ± 33.4), those who received physical therapy (62.7 ± 33.9), and those who had gastrointestinal (65.4 ± 32.5) or skin (64.9 ± 30.8) diseases (see Additional file 7). TBQ scores did not differ for the most common healthcare location (public hospital, private hospital, general practice, or specialist clinic) (*P* = 0.97).Overall, treatment burden scores were homogeneous and comparable between countries. The lowest mean (SD) TBQ score was for the item related to self-monitoring (from 0.6 ± 1.1 in Canada to 1.7 ± 2.8 in Australia/New Zealand) and the highest was for the item related to the effect of healthcare on relationships with others (from 4.0 ± 3.5 in Canada to 6.7 ± 3.8 for Australia/New Zealand) (Figure 2).

## Discussion

We adapted the TBQ for patients with one or more chronic conditions living in the USA, Canada, the UK, Australia, or New Zealand. This is the first valid and reliable instrument assessing treatment burden across multiple conditions and treatments.

During the adaptation process, we added two new items to the English TBQ that did not exist in the original study, which took place in France [9]. First, we added an item about the financial treatment burden. In France, the national health program guarantees healthcare free of charge for patients with chronic conditions. However, in countries where the English TBQ was administered, this is not always the case [10,17]. For example, 30% to 47% of patients in the USA may have problems paying medical bills [32]. Second, during the pretest, patients expressed difficulties that they had in their relationships with healthcare providers. This item was not included in the original French questionnaire because the authors felt that it measured a concept different from treatment burden. However, it was mentioned in subsequent qualitative studies [2,15,16] and, in the present study, participants insisted on including this item to take into account how discussion with physicians could affect how much they knew about their conditions and treatment, and how non-optimal relationships with healthcare providers could hinder adherence to treatment, lifestyle changes, or follow-up. During the first study, patients may have felt hindered in mentioning this issue to the investigators during face-to-face interviews and therefore the point was underestimated. Use of a web-based questionnaire has shown a decrease in socially desirable responses, and an increase in willingness to disclose sensitive information [33].

Our results highlight the advantages of using an internet platform: we were able to include 200 patients with chronic conditions from different countries in the pretest in less than 1 month. Therefore, we gathered rich qualitative data on questionnaire items and possible missing concepts, indicating the high content validity of our tool.

The item properties of the English TBQ were comparable with those from the original study in France with 1) a significant floor effect (>15%) for all items, which reflects that patients could have no burden in the aspects of their care that they have already integrated into their lives, and 2) about 30% of ‘Does not apply’ for the item related to self-monitoring. As for the original questionnaire in French, we chose to keep the item about self-monitoring because it was considered a mandatory item for other patients, particularly those with diabetes.

The validation study included 610 patients from different countries with different conditions and treatments. As hypothesized, we highlighted statistically significant associations between the overall treatment burden and adherence to medication, patients’ confidence in their knowledge about their conditions and treatment, and quality of life. To our knowledge, this is the first study to report such associations independent of disease or treatment contexts. Development of interventions to improve patient knowledge about therapeutic options could lead to shared decision making, reduced treatment burden,better adherence to medication, and better quality of life for patients.

We compared TBQ scores between countries and found that most item scores were homogeneous for patients in the USA, UK, Canada, and Australia/New Zealand, with two exceptions. First, we found increased financial treatment burden for patients residing in the USA and Australia. This result was expected because qualitative studies in these countries described the financial burden as one of the issues most widely discussed by patients [10,17]. Second, in the UK, we found increased treatment burden related to patients’ relationships with their healthcare providers. This finding could be related to pay-for-performance initiatives (for example, Quality and Outcomes Framework) that may encourage physicians to focus on biomedical objectives and spend less time listening to patients’ non-medical problems. More research is needed to understand the precise aspects of treatment burden, and why patients might experience different aspects in each of these countries.

This study has several limitations. First, to measure patients' confidence in their knowledge about their treatments and conditions, we used questions that were not validated. However, participants reviewed these questions during the pretest in the same way they reviewed the questions of the TBQ. Patients expressed no difficulties in answering these questions, and we found neither any missing answers, nor a floor or ceiling effect. Second, we recruited patients through the PLM website only, and from this patient group, only 20% of the invited patients responded to the web survey. This response rate was similar to that from other studies involving online surveys. This recruitment method might have selected patients willing to share their experiences with others and/or with severe or rare conditions. We did not include participants who were not able to access a computer. Therefore, our sample is not representative of the general population of patients with one or more chronic conditions: overall patients in our study were younger, more educated, and more often female (similar to other studies involving the PLM website [34-36]). Prevalence of chronic conditions in our study differed from those of chronic conditions of patients seeking healthcare in national surveys. For example, we found a high proportion of patients with neurological conditions, because PLM was initially developed for these patients. Although these biases were considered relatively small, psychometric properties for the TBQ (especially reliability estimates) might differ in other populations. Despite these limitations, use of an online platform has a number of advantages. First, we were able to involve a larger number of patients than in usual cross-cultural adaptation studies (800 participants including pretest) [37] in a very short time (2 months from pretest to results). Second, the recruited patients were geographically dispersed and thus, we might have avoided selecting only patients living near research centers. Finally, patients involved were engaged in research, and therefore provided us with rich feedback at every stage of our research, which may have contributed to the development of questionnaire items closer to patients’ own words and problems.

The present study advances the evidence of the validity of the TBQ. Further studies should focus on testing other psychometric properties such as responsiveness in longitudinal and prospective studies. The TBQ can be implemented in clinical trials assessing interventions intended to mitigate treatment burden and its negative effect on quality of life to develop a Minimally Disruptive Medicine [1].

## Conclusions

We adapted and validated the TBQ for English-speaking countries (USA, UK, Canada, and Australia) using an online patient-based platform for direct interaction with patients. This study resulted in the first instrument assessing treatment burdenfor any condition and treatment context in these countries.

## Competing interests

MH and PW are employees of PatientsLikeMe, and own stock or stock options in the company. The PatientsLikeMe research and development team has received research support from pharmaceutical companies and private foundations. VTT, VMM, CB and PR have no financial interests in PatientsLikeMe and no other conflicts of interest to disclose.

## Authors’ contribution

VTT, MH, VMM, CB, PW, and PR conceived and designed the study. MH and PW acquired the data. VTT and CB analyzed and interpreted the data. VTT drafted the manuscript. MH, VMM, CB, PW, and PR critically revised the manuscript for important intellectual content. PR and PW provided administrative, technical, and material support. All authors saw and approved the final manuscript. PR is the guarantor, had full access to the data in the study, and takes responsibility for the integrity of the data and the accuracy of the data analysis.

## Pre-publication history

The pre-publication history for this paper can be accessed here:

http://www.biomedcentral.com/1741-7015/12/109/prepub

## Supplementary Material

Additional file 1 Demographic and clinical characteristics of patients included in the pretest of the Treatment Burden Questionnaire (TBQ) (n = 200). Click here for file

Additional file 2 Item properties of the Treatment Burden Questionnaire (TBQ) (n = 610 patients). Click here for file

Additional file 3 Correlation of each item of the Treatment Burden Questionnaire (TBQ) with the global score, omitting that item from the total (n = 610). Click here for file

Additional file 4 Scree plots and eigenvalues for the correlation matrix of the factor analysis (n = 610). Click here for file

Additional file 5 Correlation between treatment burden scores and clinical variables in terms of treatment workload (n = 610). Click here for file

Additional file 6 Reliability testing by a test–retest method (n = 282). Click here for file

Additional file 7 Treatment Burden Questionnaire (TBQ) global score by different subgroups (n = 610). Click here for file
