# Supplementary material for: Adaptation and validation of the Treatment Burden Questionnaire (TBQ) in English using an internet platform
Source: BMC Med. 2014 Jul 2;12:109. doi: 10.1186/1741-7015-12-109 (PMC4098922; doi:10.1186/1741-7015-12-109)
Supplement: Additional file 1 — Demographic and clinical characteristics of patients included in the pretest of the Treatment Burden Questionnaire (TBQ) (n = 200). [file 1741-7015-12-109-S1.docx]

**Additional file 1: Demographic and clinical characteristics of patients included in the pretest of the English Treatment Burden Questionnaire (TBQ)** **(n=200).**

| Characteristic | Value |
| --- | --- |
| Age, yr | 49.6 (12.5) |
| Female sex – no. (%) | 141 (70.5%) |
| Country of residence – no. (%)  United States of America  Canada  United Kingdom  Australia and New Zealand  Other | 142 (71.0%)  19 (9.5%)  20 (10.0%)  8 (4.0%)  12 (6.0%) |
| No. of tablets and pills/day | 9.7 (7.9) |
| No. of injections/week | 0.7 (3.6) |
| No. of drug administrations/day | 3.1 (1.8) |
| No. of different doctors the patient regularly sees) | 3.3 (2.7) |
| No. of appointments/month | 3.1 (4.2) |
| No. of hospitalizations/year | 0.7 (2.0) |
| Presence of an informal caregiver – no. (%) | 116 (58.0%) |
| Most frequent location for medical consultations – no. (%)  Public hospital  Private hospital  General practitioner’s clinic  Specialist’s clinic | 17 (8.5%)  10 (5.0%)  94 (47.0%)  79 (39.5%) |
| Duration of oldest chronic condition – no. (%)  < 5 years  5–10 years  > 10 years | 33 (16.5%)  31 (15.5%)  136 68.0%) |
| No. of different chronic conditions | 2.9 (2.1) |
| Conditions – no. (%)*  Diabetes  Other endocrine disorders  Lung diseases  High blood pressure  Heart diseases  Kidney diseases  Gastro intestinal diseases  Stroke or cerebrovascular diseases  Neurologic diseases  Rheumatologic diseases  Cancer or malignant blood diseases  Psychiatric diseases  Vision problems  Hearing problems  Skin diseases  Infectious diseases  Fibromyalgia | 20 (10.0%)  36 (18.0%)  29 (14.5%)  48 (24.0%)  15 (7.5%)  12 (6.0%)  41 (20.5%)  9 (4.5%)  110 (55.0%)  64 (32.0%)  6 (3.0%)  95 (47.5%)  35 (17.5%)  18 (9.0%)  20 (10.0%)  3 (1.5%)  22 (11.0%) |

Data are mean (SD) unless indicated. *A given patient can have multiple conditions
