# Supplementary material for: Adaptation and validation of the Treatment Burden Questionnaire (TBQ) in English using an internet platform
Source: BMC Med. 2014 Jul 2;12:109. doi: 10.1186/1741-7015-12-109 (PMC4098922; doi:10.1186/1741-7015-12-109)
Supplement: Additional file 2 — Item properties of the Treatment Burden Questionnaire (TBQ) (n = 610 patients). [file 1741-7015-12-109-S2.docx]

**Additional file 2: Item properties of the TBQ (n=610 patients).**

| Item | Response rate  No. (%) | Item  “Does not apply”  No. (%) | “No burden”  (score=0)  No. (%) | “High burden” (score >7)  No. (%) |
| --- | --- | --- | --- | --- |
| The taste, shape or size of your tablets and/or the annoyances caused by your injections (e.g., pain, bleeding, bruising or scars) | 607 (99.5%) | 44 (7.2%) | 217 (35.6%) | 95 (15.6%) |
| The number of times you should take your medication daily | 606 (99.3%) | 23 (3.8%) | 184 (30.2%) | 55 (9.0%) |
| The efforts you make not to forget to take your medications (e.g., managing your treatment when you are away from home, preparing and using pillboxes) | 606 (99.3%) | 19 (3.1%) | 182 (29.8%) | 85 (13.9%) |
| The necessary precautions when taking your medication (e.g., taking them at specific times of the day or meals, not being able to do certain things after taking medications such as driving or lying down) | 607 (99.5%) | 28 (4.6%) | 171 (28.0%) | 115 (18.8%) |
| Lab tests and other exams (e.g., blood tests or radiology): frequency, time spent and associated nuisances or inconveniences | 608 (99.7%) | 24 (3.9%) | 173 (28.4%) | 98 (16.1%) |
| Self-monitoring (e.g., taking your blood pressure or checking your blood sugar): frequency, time spent and associated nuisances or inconveniences | 604 (99.0%) | 185 (30.3%) | 214 (35.1%) | 43 (7.0%) |
| Doctor visits and other appointments: frequency and time spent for these visits and difficulties finding healthcare providers | 607 (99.5%) | 12 (2.0%) | 151 (24.7%) | 152 (24.9%) |
| The difficulties you could have in your relationships with healthcare providers (e.g., feeling not listened to enough or not taken seriously) | 606 (99.3%) | 4 (0.6%) | 171 (28.0%) | 189 (31.0%) |
| Arranging medical appointments and/or transportation (doctors visits, lab tests and other exams) and reorganizing your schedule around these appointments | 606 (99.3%) | 9 (1.5%) | 166 (27.2%) | 151 (24.7%) |
| The administrative burden related to healthcare (e.g., all you have to do for hospitalizations, insurance reimbursements and/or obtaining social services) | 607 (99.5%) | 44 (7.2%) | 147 (24.1%) | 136 (22.3%) |
| The financial burden associated with your healthcare (e.g., out-of-pocket expenses or expenses not covered by insurance) | 608 (99.7%) | 31 (5.1%) | 101 (16.6%) | 263 (43.1%) |
| The burden related to dietary changes (e.g., avoiding certain foods or alcohol, having to quit smoking) | 608 (99.7%) | 45 (7.4%) | 172 (28.2%) | 152 (24.9%) |
| The burden related to doctors' recommendations to practice physical activity (e.g., walking, jogging, swimming) | 608 (99.7%) | 35 (5.7%) | 155 (25.4%) | 153 (25.1%) |
| How does your healthcare impact your relationships with others (e.g., being dependent on others and feeling like a burden to them, being embarrassed to take your medications in public) | 607 (99.5%) | 18 (2.9%) | 112 (18.4%) | 240 (39.3%) |
| 'The need for medical healthcare on a regular basis reminds me of my health problems’ | 607 (99.5%) | - | 71 (11.6%) | 295 (48.4%) |
