# Supplementary material for: Adaptation and validation of the Treatment Burden Questionnaire (TBQ) in English using an internet platform
Source: BMC Med. 2014 Jul 2;12:109. doi: 10.1186/1741-7015-12-109 (PMC4098922; doi:10.1186/1741-7015-12-109)
Supplement: Additional file 4 — Scree plots and eigenvalues for the correlation matrix of the factor analysis (n = 610). [file 1741-7015-12-109-S4.docx]

**Additional file 4: Scree plots and Eigenvalues for the correlation matrix of the factor analysis (n=610).** “Does not apply” was considered the lowest possible score (0).

| 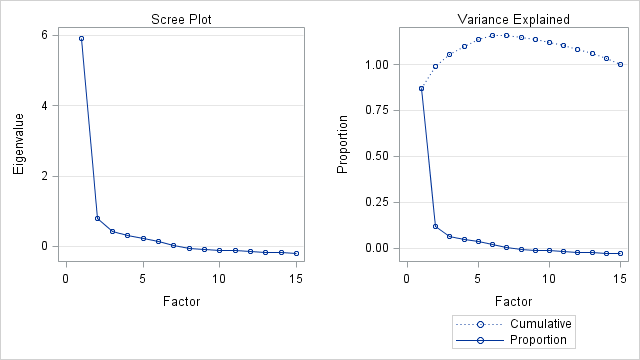 |
| --- |
|  |
| \| Factor \| Eigenvalue \| Proportion of variance explained \| Cumulative proportion of variance explained \| \| --- \| --- \| --- \| --- \| \| 1 \| 5.91270808 \| 0.8716 \| 0.8716 \| \| 2 \| 0.80393808 \| 0.1185 \| 0.9901 \| |
