# Supplementary material for: Adaptation and validation of the Treatment Burden Questionnaire (TBQ) in English using an internet platform
Source: BMC Med. 2014 Jul 2;12:109. doi: 10.1186/1741-7015-12-109 (PMC4098922; doi:10.1186/1741-7015-12-109)
Supplement: Additional file 5 — Correlation between treatment burden scores and clinical variables in terms of treatment workload (n = 610). [file 1741-7015-12-109-S5.docx]

**Additional file 5: Correlation between variables and TBQ global score (n=610)**.

| Clinical variables | Correlation with TBQ global score* | TBQ global score Mean (SD) | P value |
| --- | --- | --- | --- |
| Educational level  High school diploma or below (n=123)  College (n=478) | - | 59.1 (32.4)  51.8 (31.2) | 0.02 |
| No. of different chronic conditions  1 (n=181)  2-3 (n=234)  >4 (n=195) | - | 44.3 (29.1)  49.7 (29.0)  65.4 (33.0) | <0.0001 |
| No. of tablets and pills/day | 0.20 |  | <0.0001 |
| No. of injections/week | 0.11 |  | 0.02 |
| No. of drug administrations/day | 0.25 |  | <0.0001 |
| No. of different doctors the patient regularly sees | 0.21 |  | <0.0001 |
| No. of appointments/month | 0.25 |  | <0.0001 |
| No. of hospitalization/year | 0.11 |  | 0.01 |

*Correlations were assessed by Spearman’s correlation coefficient. Comparisons between groups involved ANOVA.
