# Supplementary material for: Adaptation and validation of the Treatment Burden Questionnaire (TBQ) in English using an internet platform
Source: BMC Med. 2014 Jul 2;12:109. doi: 10.1186/1741-7015-12-109 (PMC4098922; doi:10.1186/1741-7015-12-109)
Supplement: Additional file 7 — Treatment Burden Questionnaire (TBQ) global score by different subgroups (n = 610). [file 1741-7015-12-109-S7.docx]

**Additional file 7: TBQ global score by different subgroups (n=610).**

| Subgroup | Global TBQ score  Mean (SD) |
| --- | --- |
| Location of most frequent medical consultations  Public hospital (n=61)  Private hospital (n=19)  General practitioner’s clinic (n=286)  Specialist’s clinic (n=220) | 55.2 (33.1)  52.9 (38.6)  53.2 (30.3)  53.6 (32.0) |
| Types of treatment*  Equipment (e.g., wheelchair, walking cane) (n=80)  Physical therapy (n=98)  Over the counter drugs (n=256)  Diet (n=101)  Exercises (n=159)  Complementary and alternative medicines (n=39)  Psychotherapy (n=219)  Supplements (e.g., vitamins) (n=399)  Prescription drugs (n=545) | 69.0 (33.4)  62.7 (33.9)  59.1 (31.1)  58.1 (32.6)  56.3 (33.3)  55.8 (36.5)  55.4 (33.3)  54.3 (31.5)  54.0 (31.3) |
| Chronic conditions*  Gastrointestinal diseases (n=128)  Skin diseases (n=68)  Fibromyalgia (n=77)  Lung diseases (n=90)  Rheumatologic diseases (n=201)  Psychiatric diseases (n=245)  Diabetes (n=42)  Other endocrine disorders (n=119)  Heart diseases (n=34)  Kidney diseases (n=37)  Vision problems (n=83)  Cancer or malignant blood diseases (n=30)  Hearing problems (n=48)  High blood pressure (n=153)  Neurologic diseases (n=270)  Infectious diseases (n=18)  Stroke or cerebrovascular diseases (n=17) | 65.4 (32.5)  64.9 (30.8)  64.7 (32.2)  64.3 (35.0)  62.2 (31.7)  61.3 (32.7)  60.1 (35.6)  57.8 (32.8)  57.8 (38.7)  57.7 (36.8)  57.5 (36.0)  57.4 (36.3)  55.9 (30.4)  51.9 (31.3)  51.8 (30.1)  51.2 (28.1)  50.3 (39.2) |

*A given patient can have multiple types of treatment and multiple conditions.
